# Study on the Durability of Acid Rain Erosion-Resistant Asphalt Mixtures

**DOI:** 10.3390/ma15051849

**Published:** 2022-03-01

**Authors:** Jiatuo Wei, Quansheng Chen, Jiaying Du, Kefei Liu, Kang Jiang

**Affiliations:** 1 School of Civil Engineering, Central South University of Forestry & Technology, Changsha 410004, China; 20201200489@csuft.edu.cn (J.W.); 20193353@csuft.edu.cn (J.D.); 2 Guangzhou Highway Engineering Group Co., Ltd., Guangzhou 510030, China; 20181100329@csuft.edu.cn; 3 Hunan Communications Research Institute Co., Ltd., Changsha 410015, China

**Keywords:** road engineering, acid rain erosion, mixture type, periodic dry-wet cycle immersion test, epoxy asphalt mixture, adhesion, pavement durability

## Abstract

Acid erosion can accelerate the process of early damage of asphalt pavement and decrease the durability of asphalt pavement. However, there are limited research results for asphalt mixtures that can resist acid rain erosion. To systematically evaluate the impact and action law of acid rain erosion on the durability of asphalt mixtures, three gradation schemes were used: periodic dry–wet cycle immersion test, contact angle test and road performance test. The acid rain erosion resistance of epoxy asphalt mixture, SBS-modified asphalt mixture and 70# matrix asphalt mixture were tested from three aspects of anti-aging performance, freeze–thaw cycle performance and fatigue performance. The results show that the erosion of acid rain can significantly decrease the adhesion between asphalt and aggregate, and affects the road performance of the asphalt mixture. Acid rain erosion can significantly decrease the mechanical properties, adhesion and durability of asphalt mixtures. Epoxy asphalt has better physical properties, adhesion and acid rain erosion resistance than 70# matrix asphalt and SBS-modified asphalt. Epoxy asphalt has excellent adhesion due to its polar group, high cohesion and thermosetting resin with low shrinkage, which can effectively resist moisture erosion, spalling and temperature stress cracking, thereby effectively resisting the erosion of acid rain. Epoxy asphalt mixture has the strongest acid rain erosion resistance, which can be further enhanced when used together with waste rubber powder and modified bamboo fiber. On the whole, asphalt mixture with high-density structure and thicker asphalt film can effectively resist acid rain erosion. The durability of asphalt concrete (AC)-type gradation mixture and stone mastic asphalt (SMA)-type gradation mixture are equivalent, and both are superior to open-graded friction courses (OGFC)-type gradation mixture. The gradation of asphalt mixtures and the type of asphalt binder have great influence on their acid rain erosion resistance and durability. In order to realize the directional control of the acid rain erosion resistance and durability of different asphalt mixtures, a multi-parameter comprehensive assessment indicator system between the type and property of asphalt, the gradation of asphalt mixture, and the acid rain resistance and durability of the mixture need to be established in the future.

## 1. Introduction

In recent years, China’s economy has achieved rapid development, and a large amount of fossil fuels such as coal and oil has been consumed. These fossil fuels produce a large amount of sulfur oxides and nitrogen oxides in the combustion process, which increases the acidity of the atmosphere and leads to serious acid rain in China [1,2,3]. On the other hand, due to the unique advantages of service performance, high-grade highways usually choose asphalt pavement. However, in the practical application process, asphalt pavement often shows obvious early damage. The durability of asphalt pavement attracted the attention of many scholars at home and abroad. Al-hasan et al. [4] used recycled coarse aggregate and polymer to prepare environmentally friendly pavement, which effectively improved the durability of asphalt pavement. Kadhim et al. [5] used animal ashes in different proportions to replace limestone to prepare asphalt mixture. It was found that when 20% animal ashes were used to replace limestone, the mechanical properties of the asphalt mixture were improved, so as to improve the durability. Existing research results [6,7] show that moisture damage is the main cause of asphalt pavement failure, and much research at home and abroad has focused primarily on the traditional moisture stability of asphalt mixtures. In fact, the durability of asphalt pavement exposed to natural environment are easily affected by acid rain.

Studies have shown that acid rain can enter the asphalt mixture through the gap to cause erosion and decrease the performance of the road. Hernandez [8], Gu [9] and Yang et al. [10] used microscopic testing technology to track and analyze the changes in the main components of asphalt mastic, and proposed corresponding reaction models according to the different components of the products. Wang et al. [11] used acid solutions with different pH value to simulate the erosion of acid precipitation on mastic, and proved that the increase of acidity of acid solution can accelerate the decrease rate of shear strength of the mastic test specimen. Eyssautier et al. [12] found that acid rain with different acidity has significant effect on the voidage and asphalt integrated road performance. Pang et al. [13] proved that the content of H+ and salts in the solution are the main determinants affecting the rheological properties of asphalt in the process of moisture damage of asphalt pavement. Zhang’s [14] research shows that acid rain has adverse affects on service performance of asphalt pavements. Zeng et al. [15] demonstrated that the erosion of acid rain led to the denudation of asphalt from the aggregate surface, resulting in the loss of alkaline components in the mineral aggregate. It can be seen that acid erosion accelerates the moisture damage process of asphalt pavement and decreases the durability of asphalt pavement. However, at present, there are few studies on the deterioration mechanism of acid rain erosion on asphalt pavement durability, and research on the acid erosion deterioration inhibition technology of asphalt pavement is still non-existent.

Epoxy asphalt is a kind of thermosetting polymer material composed of component A (epoxy resin) and component B (asphalt with curing agent); it has the characteristics of good thermal stability, strong resistance to deformation and excellent fatigue resistance, and can bond well with aggregates [16,17]. Meanwhile, epoxy asphalt has strong erosion resistance and chemical stability, can effectively resist the erosion of multiple media such as acid, alkali and salt, and has good durability [18]. In addition, the low-temperature performance of the asphalt mixture is effectively improved after adding fibers and rubber particles, thereby increasing the durability of the mixture [19].

According to the research experience of the research group, based on a series of laboratory tests, the effects of material composition, aggregate gradation and other factors on the durability of asphalt mixture can be systematically analyzed [20]. Acid erosion can accelerate the process of early damage of asphalt pavement and decrease the durability of asphalt pavement. However, there are few research results of asphalt mixture that can resist acid rain erosion. To develop an acid rain erosion-resistant asphalt mixture, and to systematically evaluate the impact and action law of acid rain erosion on the durability of asphalt mixtures, in this paper, epoxy asphalt is used as binder, and waste rubber powder and modified bamboo fiber are used as filler to prepare asphalt concrete (AC)-type dense-gradation asphalt mixture. Stone mastic asphalt (SMA) and open-graded friction course (OGFC) mixtures were obtained as the control group. A periodic dry–wet cycle immersion test is used to simulate the acid rain environment and explore the influence of different acidic environments on the adhesion performance of each asphalt and the durability of each asphalt mixture, and grey relational analysis is used to analyze the contributing elements for the durability of the asphalt mixture under an acid environment. The research results can be used for the development and application of acid rain erosion-resistant asphalt mixtures.

## 2. Materials and Methods

### 2.1. Materials

In the early stage of this study, epoxy asphalt, tafpack-super (TPS) high-viscosity asphalt, styrene–butadiene–styrene (SBS) block copolymer modified asphalt and 70# matrix asphalt were selected as asphalt binders, and waste rubber powder, lignin fiber, reed fiber and modified bamboo fiber were selected as fillers to prepare dense asphalt mixture. However, for adhesion performance, the freeze–thaw cycle durability of the asphalt mixture, raw material source, environmental protection and preparation cost were considered. Finally, the optimal scheme of synergistic compounding of epoxy asphalt, waste rubber powder and modified bamboo fiber was optimized to prepare acid rain corrosion resistant asphalt mixture.

#### 2.1.1. Asphalt

Epoxy asphalt, SBS-modified asphalt and 70# matrix asphalt were used for experimental research, their basic properties are shown in Table 1. Among them, epoxy asphalt was composed of component A (containing epoxy resin) and component B (containing asphalt and curing agent), which was produced by ChemCo System Company (Newark, NJ) in the United States.

#### 2.1.2. Aggregates and Gradation Composition Design

**Figure 1:**
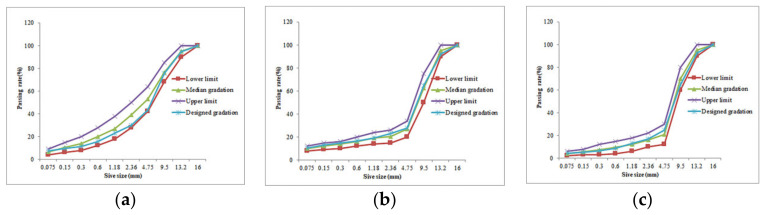
Gradation curve of each asphalt mixture. (**a**) AC-13, (**b**) SMA-13 and (**c**) OGFC-13.

Adopting limestone as the coarse aggregate of mixture, limestone chips as the fine aggregate of mixture and limestone powder as the filler, all aggregates were produced in Hengyang Quarry (Hengyang, China). To analyze the acid erosion resistance of different types of mixture, three gradations of AC-13, SMA-13 and OGFC-13 were designed respectively, and the gradation composition is shown in Table 2, the gradation curve is shown in Figure 1.

#### 2.1.3. Waste Rubber Powder and Modified Bamboo Fiber

Waste rubber powder and modified bamboo fiber were added to the asphalt mixture to enhance the adhesion effect between asphalt and aggregate, thereby improving the road performance of mixture. Among them, waste rubber powder comes from waste truck tire with the particle fineness of 80 mesh, and its content is 2% of the mass of the asphalt binder. Modified bamboo fiber is a flocculent fiber made of moso bamboo, its length is 1000–2500 μm, the relative density is 0.90–0.94, the moisture content is less than 3% and its content is 0.4% of the mass of the mixture.

### 2.2. Experiments

#### 2.2.1. Physical Properties of Asphalt Binder

Performance testing of asphalt binders included conventional performance and adhesion performance. To evaluate the adhesion performance of each asphalt binder, DSA100 contact angle measuring instrument produced by KRUSS (Hamburg, Germany) was used for the contact angle test, by testing the contact angle between known liquid and each asphalt under different acid solution immersion periods at 25 °C, the surface free energy *γ* and its dispersion component *γ*^d^ and polar component *γ*^p^ of asphalt were calculated according to surface free energy theory. The test liquids were distilled water, glycerol and formamide.

#### 2.2.2. Preparation of Asphalt Mixture

The preparation of the asphalt mixture involved a number of steps. (1) Waste rubber powder was added to SBS-modified asphalt, epoxy asphalt component B and 70# matrix asphalt, heated to 180 °C, respectively, then sheared and swelled to prepare the waste rubber powder for the asphalt. (2) Aggregates and modified bamboo fiber were preheated and poured into a mixing pot for pre-mixing. (3) A certain amount of waste rubber powder with modified asphalt was placed into the pot for mixing. For the epoxy asphalt mixture, component A was also poured into the mixing pot to continue mixing. (4) The preheated mineral powder and mix were added evenly to obtain the corresponding asphalt mixture. The composition and basic characteristics of each asphalt mixture are shown in Table 3.

#### 2.2.3. Periodic Dry–Wet Cycle Immersion Test

In order to simulate the actual composition of acid rain, concentrated sulfuric acid and concentrated nitric acid with a concentration of 98% were used to prepare the acid solution with the molar ratio c(SO_4_^2−^): C (NO_3_^−^) = 9:1. Distilled water with pH = 7 was used as the main component of the soaking liquid, and soaking liquids with pH = 2, pH = 4.5 and pH = 7 were prepared, respectively. The periodic dry–wet cycle immersion test was carried out for each asphalt mixture sample, with acid soaking for 6 days and natural drying for 1 day as a cycle, and the pH value remained constant throughout the whole test cycle.

#### 2.2.4. Aging Test 

The aging performance of the mixture after soaking was tested in different aging times and different acid pH values. An oven to heat and age different asphalt mixtures at 85 °C was used, and the size of the specimen was 300 mm × 300 mm × 50 mm. When the test condition was pH = 7, the aging time was 0 h, 30 h, 60 h, 90 h and 120 h, respectively. When the aging time was 120 h, the pH value of the acid solution was pH = 2, pH = 4.5 and pH = 7, respectively.

#### 2.2.5. Low-Temperature Stability Test

The low-temperature stability of the asphalt mixture is characterized by flexural stiffness modulus and maximum flexural tensile strain [21], and the trabecular bending test was adopted. The size of prism trabeculae was 250 mm × 30 mm × 35 mm, the test temperature was −10 °C and the loading rate was 50 mm/min.

#### 2.2.6. Moisture Stability Test 

The moisture stability of the asphalt mixture was evaluated by immersion using the Marshall test and freeze–thaw splitting test, and the immersion Marshall residual stability and freeze–thaw splitting strength of asphalt mixture were measured respectively [22]. The immersion Marshall test and freeze–thaw splitting test were used to evaluate the moisture stable aging durability, and freeze–thaw splitting test under different freeze–thaw cycles were used to evaluate the freeze–thaw cycle durability. According to the test procedure JTG E20-2011, the Marshall test specimens were divided into two groups. The Marshall stability *S*_1_ of one group was measured after curing for 0.5 h in 60 °C water bath, and the Marshall stability *S*_2_ of the other group was measured after constant temperature curing in the same conditions for 48 h. The ratio of *S*_2_*/S*_1_ was calculated as residual stability *S_0_*.

#### 2.2.7. Fatigue Life Test 

A four-point trabecular bending fatigue test was used for the fatigue life of the asphalt mixture [23]. The size of prism trabeculae was 380 mm × 63 mm × 50 mm, the test temperature was 15 °C, the frequency control was 10 Hz, and the tensile strain levels were 0.3, 0.4 and 0.5, respectively. 

**Figure 2:**
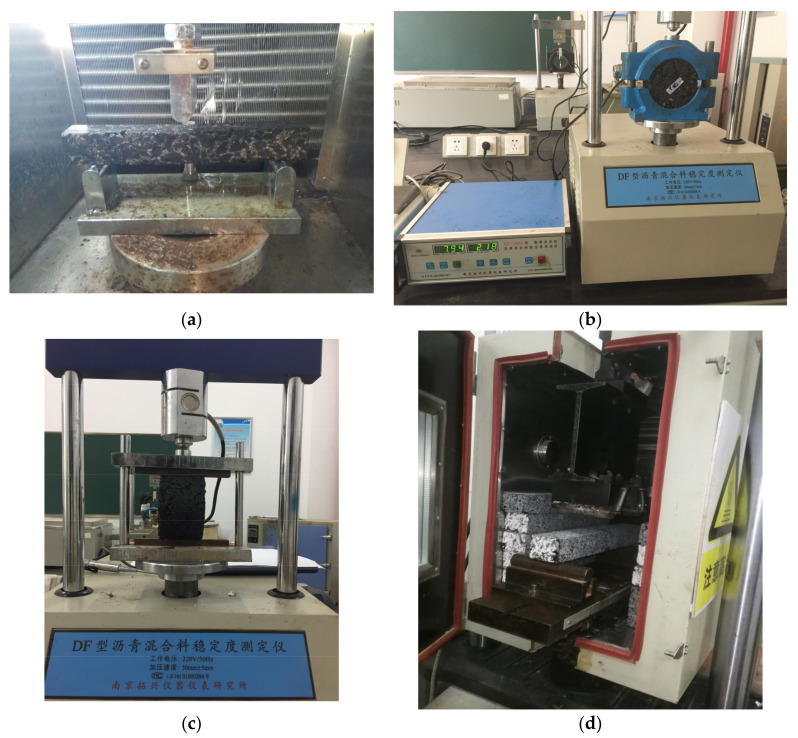
Diagram of each test device. (**a**) Trabecular bending test, (**b**) Marshall test, (**c**) splitting strength test and (**d**) four-point trabecular bending fatigue test.

Each test device is shown in Figure 2.

## 3. Results and Discussion

### 3.1. Performance Analysis of Asphalt Binder

#### 3.1.1. Physical Performance

Table 4 is the physical performance test results of each asphalt binder.

Table 4 shows that the softening point of epoxy asphalt is 11.9% and 93.1% higher than that of SBS-modified asphalt and 70# matrix asphalt, while the penetration decreased by 19.8% and 47.7%, respectively, indicating that epoxy asphalt has higher consistency, good stiffness and deformation resistance. This is due to the epoxy resin system in epoxy asphalt playing a fixed role in the asphalt. The aromatic ring contained in the curing molecules in epoxy asphalt provides a certain strength, and the reactants are superimposed on each other to form a three-dimensional interactive network structure, which gives the epoxy asphalt superior resistance to deformation [24]. The ductility of epoxy asphalt is 31.3% and 79.8%, exceeding the SBS-modified asphalt and 70# matrix asphalt, respectively. This is because epoxy asphalt maintains partial viscous characteristics in a low-temperature environment, so it has better low-temperature crack resistance [25]. Among the three types of asphalt, epoxy asphalt has the highest dynamic viscosity because the cross-linking network formed by the curing reaction of the epoxy resin system has the characteristics of gelation. Therefore, epoxy asphalt has better adhesion [26]. Compared with SBS-modified asphalt, the viscosity and toughness of epoxy asphalt are increased by 11.5% and 6.8%, respectively, and the corresponding increase of 70# matrix asphalt is 69.8% and 51.6%, showing that the epoxy asphalt system has strong cohesion, dense molecular structure and better sticky toughness.

#### 3.1.2. Adhesion Performance

**Figure 3:**
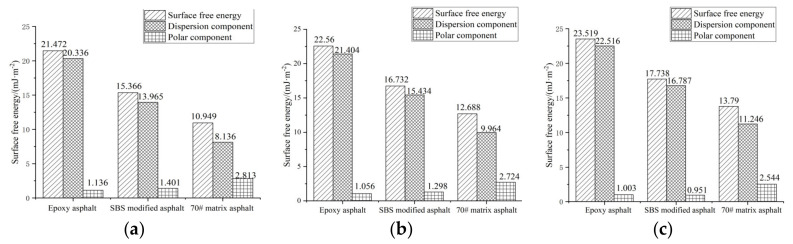
Adhesion performance of each asphalt binder. (**a**) pH = 2, (**b**) pH = 4.5 and (**c**) pH = 7.

The surface free energy of asphalt (composed of a dispersion component and polar component) is the embodiment of intermolecular force on the surface of asphalt binder; that is, the larger the value, the better the adhesion of the asphalt binder [27]. Figure 3 is the adhesion performance test results of each asphalt binder. 

Figure 3 shows that compared with SBS-modified asphalt and 70# matrix asphalt, epoxy asphalt has high surface free energy. This is because the epoxy group and hydroxyl group contained in an epoxy asphalt system have great activity, so that the epoxy cured product has excellent adhesion performance, thus improving the adhesion performance of epoxy asphalt [28].

Compared with a neutral environment (pH = 7), when pH = 4.5, the surface free energy of epoxy asphalt, SBS-modified asphalt and 70# matrix asphalt decreased by 4.10%, 5.67% and 7.99%, respectively; when pH = 2, the corresponding decrease of surface free energy of the three asphalts are 8.70%, 13.37% and 20.60%, respectively. It demonstrates that the erosion of acid rain can decrease the adhesive property of asphalt, and the moisture absorption performance of the interface between asphalt and aggregates will increase after acid rain erosion, and the asphalt film is easier to fall off from the surface of aggregates [29]. In addition, the decrease of surface free energy of epoxy asphalt is the smallest under various acidic conditions, showing that it has excellent acid erosion resistance. According to existing research results [30], the surface free energy of asphalt binder directly affects the splitting strength and freeze–thaw splitting strength of the asphalt mixture, and further affects the moisture stability of the mixture. Therefore, the epoxy asphalt mixture has better moisture stability. 

### 3.2. Anti-Aging Properties of Asphalt Mixture

#### 3.2.1. Different Aging Time

**Figure 4:**
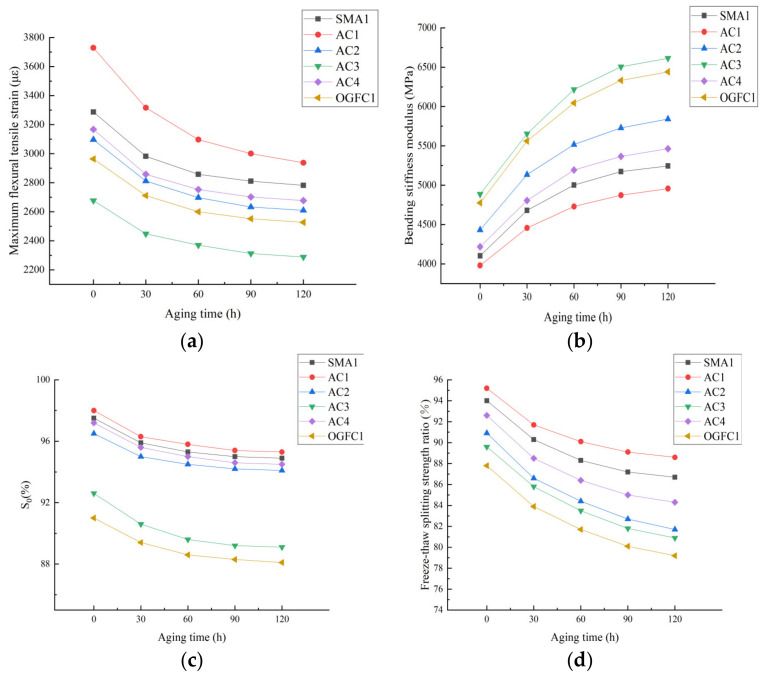
Low temperature performance and moisture stability of each asphalt mixture under different aging times. (**a**) Maximum flexural tensile strain, (**b**) bending stiffness modulus, (**c**) immersion Marshall residual stability and (**d**) freeze–thaw splitting strength ratio.

Figure 4 shows the test results of the low temperature performance and moisture stability of each asphalt mixture under different aging times.

According to Figure 4 we can establish the following:

(1) Aging has an adverse effect on the road performance of asphalt mixtures. The reason is that aging leads to an increase in the proportion of large molecular weight components in asphalt [31], and the asphalt becomes hard and brittle, causing the low-temperature cracking resistance of the asphalt mixture to be reduced. During the aging process of asphalt, ketones, strong hydrophilic substances such as ketones and surfactants will be produced; so that more asphalt molecules are dissolved in water, the porosity of the mixture increases, and the moisture damage of the asphalt mixture is aggravated [32].

(2) Under the same gradation, the low-temperature performance and moisture stability of the epoxy asphalt mixture are better than for other asphalt mixtures, indicating that the epoxy asphalt mixture has stronger resistance to low-temperature cracking and moisture loss after aging. This is because epoxy asphalt has better low-temperature creep properties, and its adhesion with aggregates is better than SBS-modified asphalt and 70# matrix asphalt [33].

(3) Under a certain aging time, the road performance of asphalt mixtures are ranked as AC1 > SMA> AC4 > AC2 > OGFC > AC3. The main reason for this ranking is that the fine aggregate content in the AC-type mixture is higher, and the asphalt binder can better wrap the aggregate particles, so the AC-type mixture has stronger anti-aging and low-temperature deformation resistance. In addition, the asphalt mixture containing waste rubber powder and modified bamboo fiber has better aging durability, mainly because the addition of waste rubber powder can physically and chemically react with the asphalt to form a gel and melt expansion structure, while the vertical and horizontal distribution of bamboo fiber can play a role of crack resistance and shrinkage under the action of external force, and the combination of the two effectively improve the aging durability of mixture [20].

#### 3.2.2. Different Acidic Conditions

To analyze the influence of acid erosion on the aging durability of asphalt mixture, the asphalt mixtures were corroded in different acid solutions for 0, 1, 3 and 5 cycles, and the road performance of each mixture was tested with an aging temperature of 80 °C and after an aging time of 120 h.

##### Low-Temperature Performance

**Figure 5:**
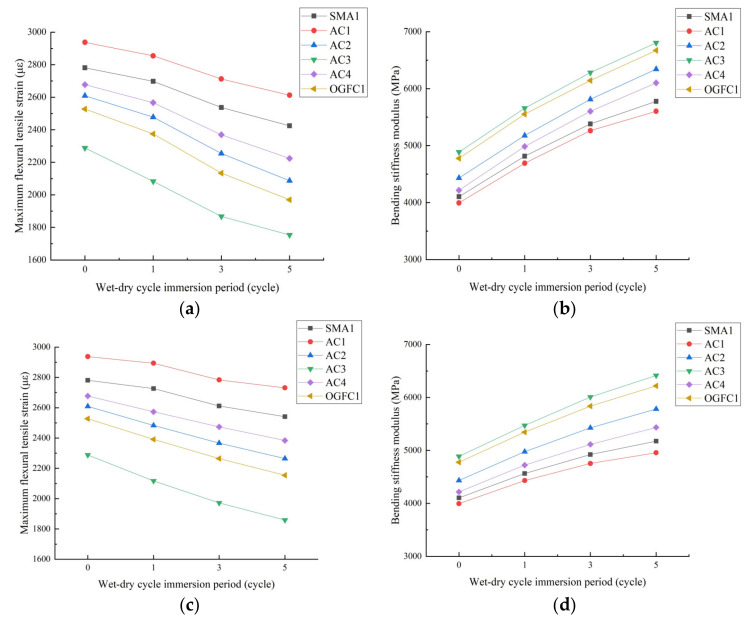

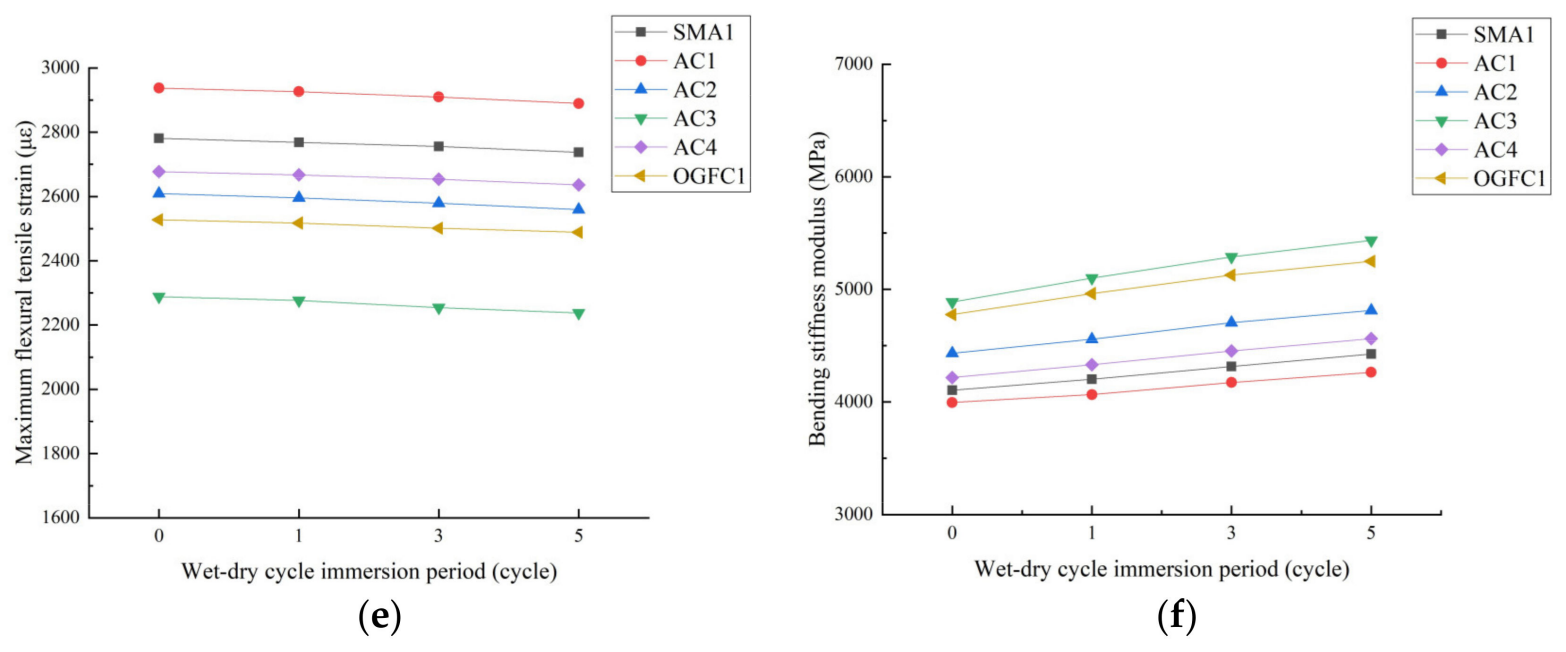
Low-temperature properties of each asphalt mixture after different acid erosion and aging. (**a**) pH = 2, (**b**) pH = 2, (**c**) pH = 4.5, (**d**) pH = 4.5, (**e**) pH = 7 and (**f**) pH = 7.

Figure 5 shows the low-temperature performance test results of each asphalt mixture with different acid erosion and aging components.

**Figure 6:**
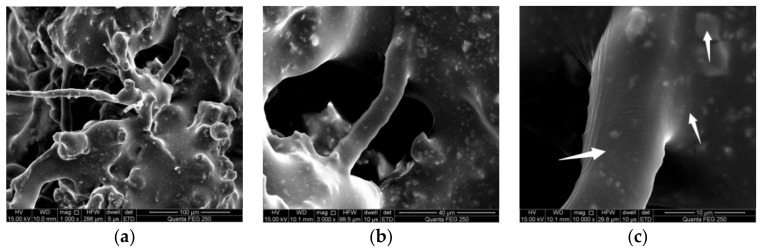
Microstructure of fiber asphalt mastic in asphalt mixture. (**a**) 1000 times, (**b**) 3000 times and (**c**) 10,000 times.

Figure 5 establishes the following:

(1) After soaking in acid solution with pH = 2 or pH = 4.5, the maximum flexural tensile strain of each asphalt mixture after aging decreased significantly with the increase of acid concentration and soaking period, and the changing trend of bending stiffness modulus is completely opposite. It shows that the acid erosion weakens the anti-aging performance of asphalt, increases the stiffness modulus, deteriorates the ductility and fluidity and further decreases the low-temperature crack resistance of asphalt mixture [29].

(2) Under the same gradation, acid concentration and soaking time, the maximum flexural tensile strain of the epoxy asphalt mixture is higher than that of other asphalt mixtures, and its bending stiffness modulus is the smallest of the three asphalt mixtures, proving that the mechanical properties of the epoxy asphalt mixture after acid erosion and aging are stronger. This is because epoxy asphalt has better acid erosion resistance [34] and low-temperature creep performance, and its bonding performance with the aggregate is better than SBS-modified asphalt and 70# matrix asphalt, thereby improving the low-temperature toughness of the asphalt mixture after erosion and aging. Furthermore, the epoxy asphalt mixture has the least change in low-temperature performance, indicating that its acid erosion resistance and low-temperature stable aging durability are the best.

(3) Under the same gradation, acid concentration and soaking time, the asphalt mixture containing waste rubber powder and modified bamboo fiber has better acid resistance and low-temperature stable aging durability, showing that the synergistic effect of waste rubber powder and modified bamboo fiber can increase the acid resistance of the asphalt mixture. Figure 6 shows the microstructure of fiber asphalt mastic in asphalt mixtures under different magnification. As can be seen, after adding waste rubber powder and modified bamboo fiber, the optimum asphalt content of asphalt mixture improved. Waste rubber powder melts and expands to absorb part of the light components in the asphalt to produce surface adhesion, and the modified bamboo fiber increases the asphalt content and the proportion of “structural asphalt” in the asphalt mixture by adsorbing free asphalt. which has a good restriction and constraint on the spalling of fine material of asphalt mastic under the action of acid erosion, thereby slowing down the erosion rate of acid solution on the asphalt mixture [35]. Meanwhile, the randomly distributed fibers can effectively delay the development of cracks in asphalt mixtures and the addition of waste rubber powder can fill the void of asphalt mixture to give it good compactness, so as to effectively prevent acid rain from entering the mixture and causing erosion.

When the acid concentration is the same, the low-temperature crack resistance of each asphalt mixture ranked from superior to inferior as AC > SMA > OGFC, indicating that the moisture stable aging durability of AC-type asphalt mixture is the best, followed by SMA-type, and OGFC-type is the worst. This is because the aggregate of AC-type mixture is finer, which can fill the internal voids of the mixture together with (fiber) asphalt mastic, thus improving the compactness and moisture stability of the mixture. On the contrary, OGFC-type mixture has large voids, which provides more space for moisture retention, and it is easier for asphalt to peel off due to the deterioration of bonding performance. 

##### Moisture Stability

**Figure 7:**
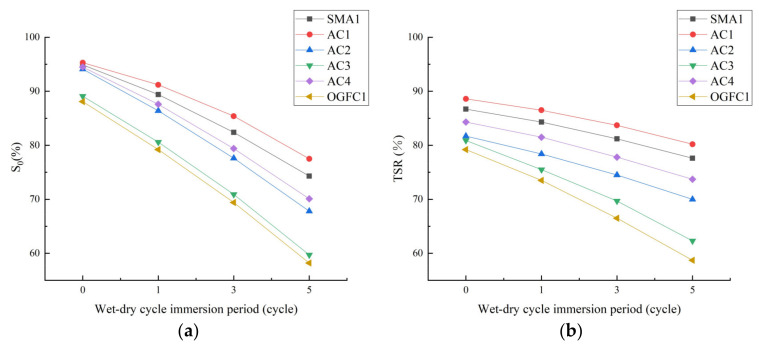

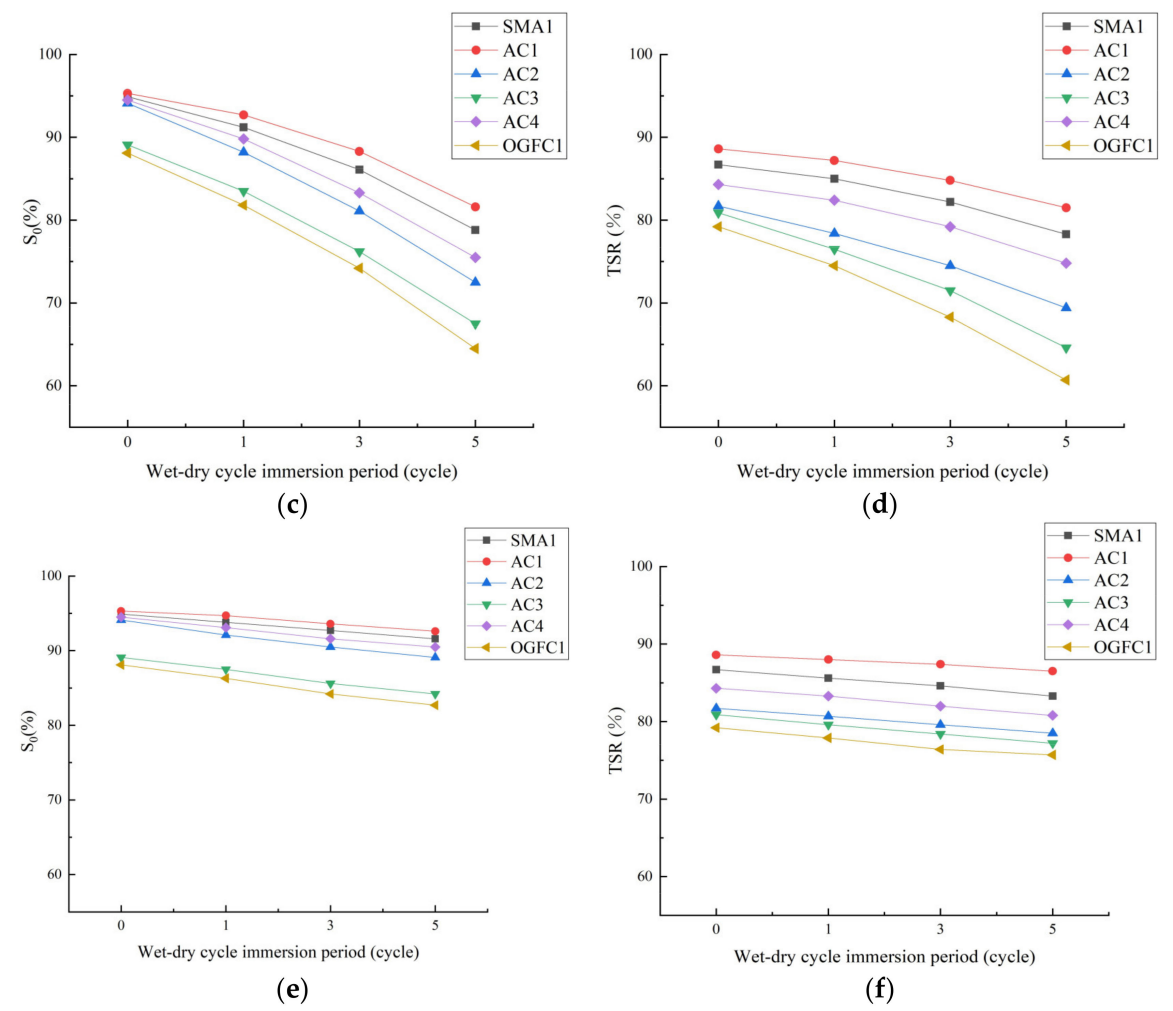
Moisture stability of each asphalt mixture after different acid erosion and aging. (**a**) pH = 2, (**b**) pH = 2, (**c**) pH = 4.5, (**d**) pH = 4.5, (**e**) pH = 7 and (**f**) pH = 7.

Researchers believe that asphalt mixture with larger *TSR* value has better moisture stability, and the *TSR* value can reflect the sensitivity of asphalt mixture to acid rain attack [7]. Figure 7 shows the test results for the moisture stability of each asphalt mixture after different acid erosion and aging.

Figure 7 establishes the following:

(1) After soaking in acid solution with pH = 2 or pH = 4.5, the *S*_0_ value and *TSR* value of each asphalt mixture show an obvious downward trend. This is mainly due to the H+ in acid solution decreases the pH value of the aggregate surface, making it more difficult for the asphaltic acid in asphalt to produce chemical adsorption with the aggregate and decrease the bonding effect of the interface between asphalt and aggregate, thereby decreasing the moisture stability of the mixture.

Under the same gradation, acid concentration and immersion period, the moisture stability of asphalt mixtures is ranked from superior to inferior as AC1 > AC4 > AC2 > AC3, demonstrating that the epoxy asphalt mixture has better moisture stability. The main reason is that polar groups in the epoxy resin curing system, such as epoxy groups, hydroxyl groups, ether bonds, amine bonds and ester bonds, give it extremely high bonding strength, and the three-dimensional network structure formed by them effectively wraps asphalt and aggregates, thus forming excellent anti-peeling strength and moisture stability.

Under the same gradation, acid concentration and immersion period, the asphalt mixture containing waste rubber powder and modified bamboo fiber has better acid resistance and moisture stable aging durability, showing that the erosion resistance and moisture stability of the asphalt mixture are improved by the synergistic effect of waste rubber powder and modified bamboo fiber. Modified bamboo fiber improves the spatial structure of asphalt mastic, and waste rubber powder improves the viscosity of asphalt and the compactness of mixture delays the penetration of moisture, so the moisture stability of the asphalt mixtures is enhanced.

### 3.3. Freeze–Thaw Cycle Durability of Asphalt Mixture

**Figure 8:**
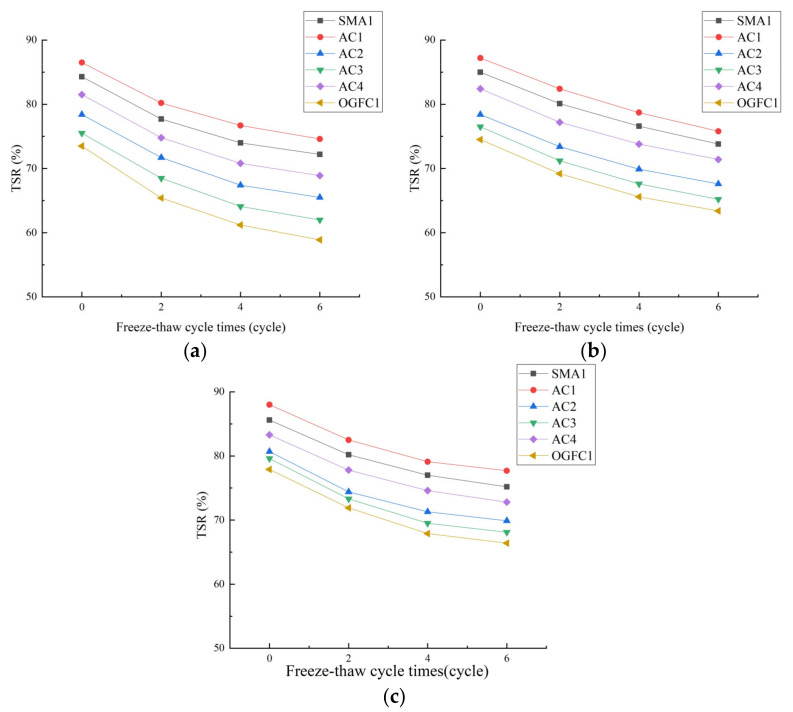
*TSR* value of each asphalt mixture under different freeze-thaw cycles. (**a**) pH = 2, (**b**) pH = 4.5, (**c**) pH = 7.

After soaking each asphalt mixture in solutions with different acidity for one cycle, it underwent 0, 2, 4 and 6 cycles of the freeze–thaw test. The *TSR* test results are shown in Figure 8.

Figure 8 establishes the following:

The *TSR* value of each asphalt mixture decreased with the increase of the number of freeze–thaw cycles. The freeze–thaw cycle refers to the alternating of freezing and thawing of moisture inside the mixture; it is not only moisture damage but also the structural damage of the mixture caused by frost heave [36]. In the process of freezing and thawing, the freezing action will form a negative temperature slope difference effect [37]. In order to decrease the strength loss caused by water expansion, part of the moisture will be discharged to the surface of the mixture through the internal connected gap of mixture, so that the connected gap keeps increasing and the internal structure of mixture is damaged. The invasion of moisture will lead to the decrease of interfacial adhesion between asphalt and aggregate. Under the combined action of moisture damage and freeze–thaw damage, the splitting strength of the mixture rapidly decreases.

As the concentration of soaking acid solution and the number of freeze–thaw cycles increases, the *TSR* value of each asphalt mixture obviously decreases, indicating that the freeze-resistance performance of the asphalt mixture obviously attenuated under the immersion of acid rain. This is because the colloid content of asphalt decreases after acid erosion, which leads to poor adhesion, and decreases the freeze–thaw cycle durability of asphalt mixture [38]. Furthermore, the alkaline components in the aggregates are lost due to chemical reaction in acid solution, resulting in increased porosity in the asphalt mixture, which is also the reason for the decrease of the freeze–thaw splitting tensile strength [39].

(2) The freeze–thaw splitting strength of the epoxy asphalt mixture is greater than that of the other two asphalt mixtures, and its decrease rate is slower, demonstrating that epoxy asphalt mixture still has superior freeze–thaw cycle durability after acid erosion. This is due to the high cohesion of epoxy asphalt itself and good adhesion with aggregates, which can effectively resist the erosion and spalling of moisture. Moreover, the curing shrinkage rate of thermosetting resin in epoxy asphalt is very small and its linear expansion shrinkage coefficient is small, so the temperature internal stress of epoxy asphalt is small and it is less prone to crack at low temperatures, which further prevents the damage of moisture to the asphalt film–mineral aggregate system, consequently, the epoxy asphalt mixture has good freeze-thaw cycle durability and can effectively prevent the erosion of acid rain.

(3) The addition of waste rubber powder and modified bamboo fiber effectively improves the freeze–thaw cycle durability of asphalt mixture, which is due to the large number and randomly dispersed rubber powder–fiber network connecting the structure into a solid whole, so the bonding between asphalt and mineral aggregate is closer and delaying the penetration of moisture. Moreover, the fiber can play the role of absorbing and adsorbing asphalt, and the structural asphalt membrane in the asphalt mixture is increased, which further enhances the impermeability of the mixture.

(4) Under the same conditions, the splitting tensile strength of the asphalt mixture with different gradation is AC > SMA > OGFC, while the attenuation rate of splitting strength shows the opposite trend, and the freeze-resistance performance of AC-type and SMA-type mixtures is close and better than that of the OGFC-type mixture. In fact, excellent freeze-resistance performance requires sufficient coarse aggregate to form a skeleton, while maintaining compactness and proper porosity [40]. The AC-type mixture is denser and the SMA-type mixture contains a large amount of coarse aggregate, so both of them have good acid erosion resistance and frost resistance. There are several voids in the OGFC-type mixture, and acid can easily enter the interior of asphalt mixture with interconnected voids and erode, thus affecting the freeze–thaw cycle durability.

### 3.4. Fatigue Property of Asphalt Mixture

Under the condition of certain stress, the fatigue characteristics of asphalt mixture are expressed by Equation (1) [23]:

(1) $$N_{f}=k\;(\frac{1}{\sigma_{0}}{)}^{n}$$

After logarithmic transformation, the following equation can be obtained:

(2) $$lgN_{f}=-nlg\sigma_{0}+lgk$$

where, $N_{f}$ is fatigue life (times), *σ*_0_ is the initial bending stress (MPa), *k* and *n* can be determined by experiment. 

**Figure 9:**
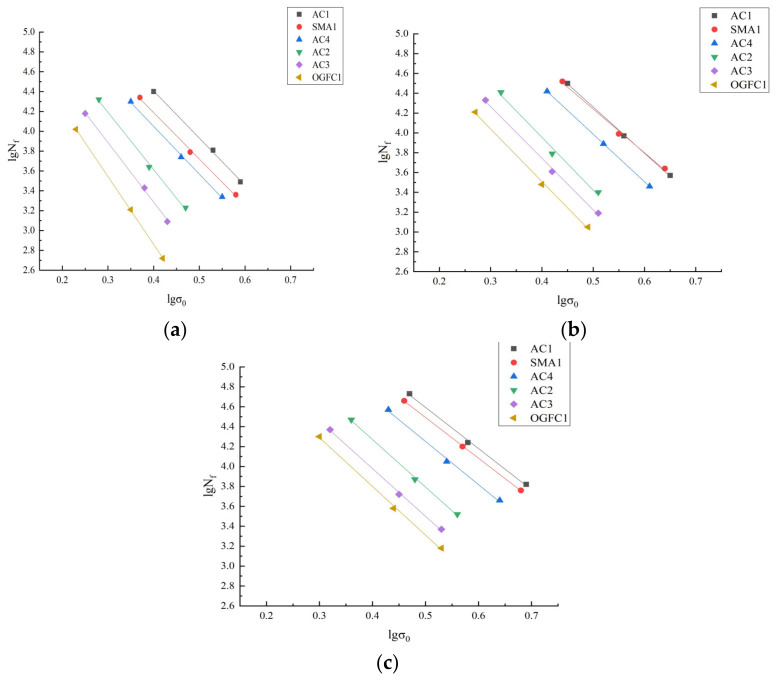
Fatigue test results for each asphalt mixture. (**a**) pH = 2, (**b**) pH = 4.5 and (**c**) pH = 7.

The four-point bending fatigue test was carried out on the trabecular bending fatigue specimens after one dry–wet immersion cycle under different acid conditions. Figure 9 shows the fatigue test results and Table 5 shows the fatigue life fitting equation.

Figure 9 establishes the following:

(1) During the use of pavement, $lgN_{f}$ and *lgσ*_0_ show a linear decreasing relationship, the asphalt mixture will produce cumulative damage due to the repeated action of climatic factors and wheel load, resulting in the decline of pavement structural strength. When the stress in the pavement exceeds the structural resistance, cracks will appear and fatigue failure will occur under the action of continuous repeated loads.

After soaking in acid solution with pH = 2 or pH = 4.5, the attenuation of the fatigue life of the asphalt mixture is more obvious. This is because the acid solution can penetrate into the interface of the asphalt-aggregate through micro-cracks to corrode the aggregate. Under the action of load stress, H^+^ further penetrates and diffuses into the mixture until the crack extends to fracture.

(2) Under a certain stress level, the epoxy asphalt mixture has the highest *k* value, and its *n* value of the fitting curve is the smallest. It shows that epoxy asphalt mixture has better fatigue durability. This is because epoxy asphalt has high bonding strength and tensile strength, which can play a certain tensile role in the separation of aggregates in the mixture. In addition, the self-healing ability of micro-cracks of epoxy asphalt is superior to SBS-modified asphalt and 70# matrix asphalt, so the epoxy asphalt mixture has excellent hysteretic recovery ability and better fatigue durability under an external load.

(3) The synergistic effect of waste rubber powder and modified bamboo fiber can give the mixture good reinforcement effect and give it better resistance to repeated loads. In addition, the addition of bamboo fiber increases the amount of structural asphalt, which is beneficial to the repair of micro-cracks, thus delaying the penetration damage of the acid solution, and improving the acid erosion resistance and fatigue property of the asphalt mixture [41]. 

(4) Under a certain stress level, the order of *k* values of asphalt mixtures with different gradations is AC1 > SMA > AC4 > AC2 > AC3 > OGFC. The ordering of *n* values is the exact reversal of *k* value, it means that the AC-type mixture has better fatigue performance after acid immersion, followed by the SMA-type mixture, and the OGFC-type mixture is the worst. This is because the fatigue life of asphalt mixtures decreases with the increase of void ratio and the decrease of cohesion [42]. In addition, the less the amount of asphalt, the smaller the cohesion between asphalt and aggregates, and the shorter the fatigue life of the mixture.

### 3.5. Grey Correlation Analysis

Grey correlation theory is employed to analyze the factors affecting the durability of the asphalt mixture after soaking in acid solution. The maximum flexural tensile strain and the *S*_0_ value are used as the evaluation indexes for the anti-aging properties of asphalt mixture, and the influencing factors are aging time, pH value of soaking solution, properties of asphalt binder, dry–wet cycle immersion period and aggregate gradation. The *TSR* value is used as the evaluation indexes for the freeze–thaw cycle durability of asphalt mixture, and the influencing factors are properties of the asphalt binder, freeze–thaw cycles, pH value of soaking solution and aggregate gradation. The fatigue life is used as the evaluation indexes for the fatigue property of the asphalt mixture and the influencing factors are properties of the asphalt binder, tensile stress level, pH value of soaking solution and aggregate gradation [20].

#### 3.5.1. Anti-Aging Properties of Asphalt Mixtures

**Figure 10:**
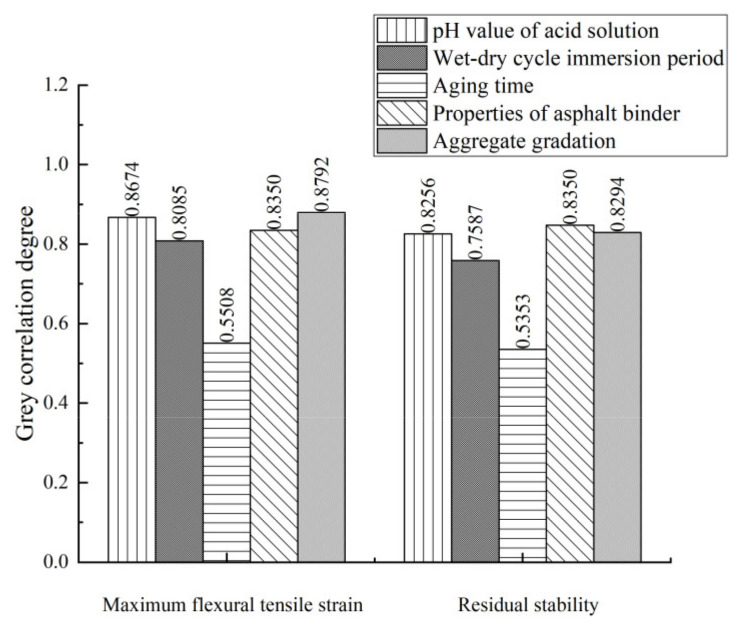
Grey correlation analysis results of influencing factors of aging durability.

Table 6 shows the evaluation indexes and influencing factors of the anti-aging properties of each asphalt mixture, and Figure 10 shows the results of grey correlation analysis.

Figure 10 shows that for low-temperature performance, the influencing factors of anti-aging properties of the asphalt mixture is arranged as aggregate gradation > pH value of soaking solution > properties of asphalt binder > aging time wet-dry cycle immersion period > aging time. The correlation between aggregate gradation and maximum flexural tensile strain is 0.8792, showing that aggregate gradation is the most influential factor for the low-temperature performance of asphalt mixtures. Good aggregate gradation is an important factor to maintain the low-temperature crack resistance of asphalt mixture after acid erosion and aging. The higher the content of fine aggregate and the smaller the porosity, the stronger the low-temperature performance of the asphalt mixture. The correlation between pH value of soaking solution and maximum flexural tensile strain is 0.8674, showing that the low-temperature performance of asphalt mixture is greatly affected by the acid concentration. There is also a high correlation between properties of the asphalt binder and the maximum flexural tensile strain. The good cohesion and erosion resistance of epoxy asphalt and its adhesion to aggregate can effectively inhibit the erosion of acid solution and the deterioration of low-temperature performance after aging.

For moisture stability, the influencing factors of anti-aging properties of asphalt mixture is arranged as properties of asphalt binder > aggregate gradation > pH value of soaking solution > wet-dry cycle immersion period > aging time. The correlation degree between properties of the asphalt binder and *S*_0_ value is 0.8479, showing that epoxy asphalt has a good effect on inhibiting the acid erosion and moisture damage of the asphalt mixture after aging. The correlation degree between aggregate gradation and *S*_0_ value is 0.8294, showing that the skeleton type, content of fine aggregates, void ratio and filling effect of fillers (waste rubber powder and modified bamboo fibers) of the asphalt mixture can directly affect the freeze–thaw resistance and moisture damage resistance of asphalt mixtures after acid erosion. The correlation degree between pH value of soaking solution and *S_0_* value is 0.8256, indicating that acid immersion can decrease the moisture stability of asphalt mixtures.

#### 3.5.2. Freeze–Thaw Cycle Durability of Asphalt Mixtures

**Figure 11:**
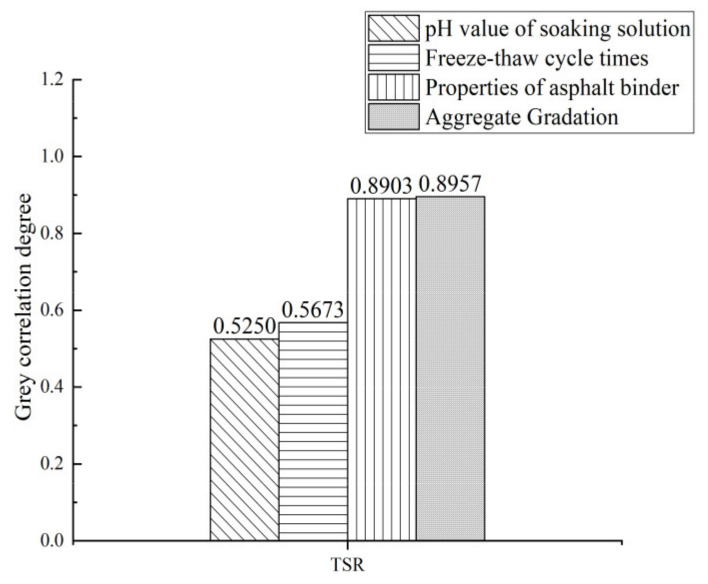
Grey correlation analysis results of influencing factors of freeze–thaw cycle durability.

Table 7 shows the evaluation indexes and influencing factors of the freeze–thaw cycle durability of the asphalt mixtures, and Figure 11 shows the result of grey correlation analysis.

Figure 11 shows that the influencing factors of the freeze–thaw cycle durability of the asphalt mixture are ranked from strong to weak as aggregate gradation > properties of asphalt binder > freeze-thaw cycle times > pH value of soaking solution. The correlation coefficient between aggregate gradation and *TSR* value is 0.8792, showing that aggregate gradation has the greatest impact on the frost resistance of asphalt mixtures. Existing studies believe that good frost resistance requires dense filling of asphalt mixtures with appropriate porosity [43]. Under the action of multiple freeze–thaw cycles, the expansion of void water causes damage to the interior of the mixture, so gradation plays a key role in ensuring the frost resistance of asphalt mixtures [44]. Asphalt binder properties have great impact on the freeze–thaw cycle durability of asphalt mixtures after acid erosion. At low temperature frost heave, the adhesion between asphalt mastic and aggregate is reduced. Epoxy asphalt effectively improves the sticky toughness and enhances the freeze–thaw cycle durability of asphalt mixtures after acid erosion.

#### 3.5.3. Fatigue Property of Asphalt Mixtures

**Figure 12:**
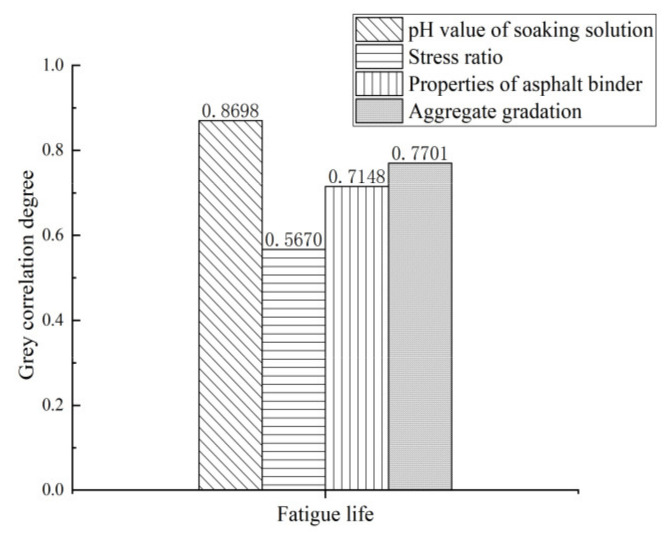
Grey correlation analysis results of fatigue property influencing factors.

Table 8 shows the evaluation indexes and influencing factors of the fatigue property of each asphalt mixture, and Figure 12 shows the results of grey correlation analysis. 

Figure 12 shows that the fatigue property influencing factors of the asphalt mixture is arranged as pH value of soaking solution > aggregate gradation > properties of asphalt binder > tensile stress level. As mentioned above, both the increase in porosity and the decrease in cohesion lead to a decrease in the fatigue life of the asphalt mixture. The less asphalt is used, the worse the cohesion between asphalt and aggregates, and the shorter the fatigue life of the mixture. The correlation coefficient between pH value of the soaking solution and fatigue life is 0.8698, showing that the erosion of acid will accelerate the structural failure of asphalt pavement, and the erosion of acid will gradually intensify as the acidity of the acid increases. 

## 4. Conclusions

A number of conclusions can be drawn from this study:

(1) After acid rain erosion, the adhesion between asphalt-aggregate and the low-temperature performance, moisture stability and fatigue performance of an asphalt mixture are significantly reduced, and the aging of the asphalt mixture is accelerated. The properties of the asphalt mixture decrease sharply with the increase of acid concentration and the extension of an immersion period.

(2) Epoxy asphalt has better physical properties, adhesion and acid rain erosion resistance than 70# matrix asphalt and SBS-modified asphalt. Epoxy asphalt has excellent adhesion due to its polar group, high cohesion and thermosetting resin with low shrinkage, which can effectively resist moisture erosion, spalling and temperature stress cracking, thereby effectively resist the erosion of acid rain.

(3) The epoxy asphalt mixture has the strongest acid rain erosion resistance, which can be further enhanced when used together with waste rubber powder and modified bamboo fiber. The addition of waste rubber powder and modified bamboo fiber increases the “structural asphalt” in the asphalt mixture, decreases the void ratio and has good compactness. The formed spatial network structure can play the role of “reinforcement” and “toughening”, thus further enhancing the durability, impermeability and acid rain erosion resistance of the mixture.

(4) On the whole, an asphalt mixture with high-density structure and thicker asphalt film can effectively resist acid rain erosion. The durability of an AC-type gradation mixture and SMA-type gradation mixture are equivalent, and both are superior to an OGFC-type gradation mixture.

(5) The gradation of asphalt mixture and the type of asphalt binder have great influence on the acid rain erosion resistance and durability of asphalt mixture. The micro-scale erosion mechanism of acid rain on different asphalt binders and mixtures is not clear; in order to realize the directional control of the acid rain erosion resistance and durability of different asphalt mixtures, a multi-parameter comprehensive assessment indicator system between the type and property of asphalt, the gradation of asphalt mixture and the acid rain resistance and durability of a mixture need to be established in the future.

## Figures and Tables

**Table 1:** Conventional properties of each asphalt.

Property	Penetration (25 °C, 0.1 mm)	Softening Point (°C)	Ductility (10 °C, cm)	Dynamic Viscosity (60 °C, Pa·s)	Mass Change after TFOT * (%)	Residual Penetration after TFOT (25 °C, %)
Epoxy asphalt	39.8	98.0	45.0	29,365	0.00	91.3
SBS-modified asphalt	49.0	87.5	34.0	24,564	−0.01	76.4
70# matrix asphalt	64.0	48.0	24.7	500	−0.02	69.8

* Note: Thin film oven test.

**Table 2:** Gradation composition of each asphalt mixture (unit: %).

Mixture Type	Passing Rate (mm)									
	16	13.2	9.5	4.75	2.36	1.18	0.6	0.3	0.15	0.075
AC-13	100	94.7	75.5	43.6	30.0	23.1	15.7	11.2	9.4	7.4
SMA-13	100	92.2	64.9	27.6	23.1	19.2	16.6	14.6	13.1	10.3
OGFC-13	100	92.3	64.6	24.8	16.9	13.0	8.9	6.4	5.4	4.3

**Table 3:** Composition of each asphalt mixture.

Mixture Type	Gradation Type	Asphalt Type			Waste Rubber Powder	Modified Bamboo Fiber	Optimum Asphalt Content (%)	Void Fraction (%)
		Epoxy Asphalt	SBS-Modified Asphalt	70# Matrix Asphalt				
AC1	AC-13	√			√	√	5.5	3.0
AC2			√		√	√	5.3	3.0
AC3				√	√	√	5.0	3.0
AC4		√					4.9	3.5
SMA	SMA-13	√			√	√	6.2	4.0
OGFC	OGFC-13	√			√	√	4.6	19.7

**Table 4:** Physical performance of each asphalt binder.

Technical Index	Epoxy Asphalt	SBS-Modified Asphalt	70# Matrix Asphalt
Softening point (°C)	98.5	88.1	51.2
Penetration (25 °C, 0.1 mm)	38.2	47.6	73.0
Ductility (10 °C, cm)	45.3	34.5	25.2
Dynamic viscosity(60 °C, Pa·s)	29,524	24,713	517
Sticky toughness(25 °C, N·m)	21.4	19.2	12.6
Toughness(25 °C, N·m)	18.8	17.6	12.4
Elastic recovery rate (%)	93	86	82

**Table 5:** The fatigue life fitting equation of each asphalt mixture.

Mixture Type	pH = 2	R^2^	pH = 4.5	R^2^	pH = 7	R^2^
AC1	y = −4.7491x + 6.3062	0.9921	y = −4.6516x + 6.58973	0.9913	y = −4.1363x + 6.66242	0.9987
AC2	y = −5.7637x + 5.9202	0.9932	y = −5.3321x + 6.08838	0.9947	y = −4.7697x + 6.17921	0.9919
AC3	y = −5.9980x + 5.6859	0.9945	y = −5.2070x + 5.82755	0.9991	y = −5.9980x + 5.68598	0.9995
AC4	y = −4.8106x + 5.9741	0.9966	y = −4.8006x + 6.38764	0.9928	y = −4.8106x + 5.97415	0.9955
SMA	y = −4.6722x + 6.0570	0.9917	y = −4.4152x + 6.44897	0.9932	y = −4.1029x + 6.54462	0.9908
OGFC	y = −6.8321x + 5.5940	0.9918	y = −5.2975x + 5.62817	0.9954	y = −4.8933x + 5.75816	0.9908

**Table 6:** Evaluation indexes and influencing factors of anti-aging properties of asphalt mixtures.

Evaluation Index	Serial Number	Maximum Flexural Tensile Strain (×10^−6^ με)	*S*_0_ (%)	Aging Time (h)	pH Value of Acid Solution	Wet-Dry Cycle Immersion Period (Cycle)	4.75 mm Pass Rate (%)	Ductility (10 °C, cm)	Dynamic Viscosity (60 °C, Pa·s)
Low-temperature stability	1	3483		0	7	1	43.6	24.7	
	2	2063		30	4.5	3	24.8	34	
	3	2307		90	2	3	27.6	45	
	4	2514		60	4.5	5	43.6	45	
	5	3014		120	7	5	27.6	24.7	
	6	1874		120	2	1	24.8	34	
Moisture stability	1		96	0	7	1	43.6		29,365
	2		91.5	30	4.5	3	24.8		24,564
	3		65.7	60	2	3	27.6		500
	4		74.2	90	4.5	5	43.6		500
	5		86.5	120	7	5	27.6		29,365
	6		87.2	120	2	1	24.8		24,564

**Table 7:** Evaluation indexes and influencing factors of freeze–thaw cycle durability of asphalt mixtures.

Serial Number	*TSR* (%)	pH Value of Acid Solution	Freeze-Thaw Cycle Times (Cycle)	Dynamic Viscosity (60 °C, Pa·s)	4.75 mm PASS Rate (%)
1	84.9	2	2	29,365	43.6
2	77.3	4.5	4	24,564	27.6
3	63.4	4.5	6	500	24.8
4	78.4	7	4	24,564	43.6
5	76.3	7	6	29,365	27.6

**Table 8:** Evaluation indexes and influencing factors of fatigue property of each asphalt mixture.

Serial Number	Fatigue Life (Times)	pH Value of Soaking Solution	Tensile Stress Level	Elastic Recovery Rate (%)	4.75 mm Pass Rate (%)
1	21,244	7	0.3	93	43.6
2	17,643	7	0.4	86	27.6
3	9477	4.5	0.5	82	43.6
4	12,737	4.5	0.3	86	27.6
5	4227	2	0.4	82	24.8
